# Influence of Porosity on Fatigue Behaviour of 18Ni300 Steel SLM CT Specimens at Various Angles

**DOI:** 10.3390/ma17020432

**Published:** 2024-01-16

**Authors:** Pablo M. Cerezo, Jose A. Aguilera, Antonio Garcia-Gonzalez, Pablo Lopez-Crespo

**Affiliations:** Department of Civil and Materials Engineering, University of Malaga, C/Dr Ortiz Ramos, s/n, 29071 Malaga, Spain; pm@uma.es (P.M.C.); j.a.aguilera@uma.es (J.A.A.); tolin@uma.es (A.G.-G.)

**Keywords:** 18Ni300, additive manufacturing (AM), fatigue, microstructure, porosity, selective laser melting (SLM)

## Abstract

In order to improve understanding of the fatigue behaviour in additive manufactured samples, this research delves into the challenging interplay between building parameters, particularly fabrication angles, and the presence of pores. The primary objective is to explore the characterisation of these pores and unravel their relationship with the fatigue properties of the material under investigation. Through a systematic analysis of porosity distribution in various fabrication orientations, supplemented by a detailed examination of the elemental dispersion around specific porous structures using energy-dispersive X-ray spectroscopy, a consistent behavioural pattern emerges across the samples. In assessing fatigue behaviour, an examination of the variables reveals that only area and aspect ratio significantly influence the behaviour of the samples. Such studies can contribute substantially to academic research in the field of material science and engineering.

## 1. Introduction

There has been a growing interest in additive manufacturing (AM) technologies in recent years as they present several potential advantages over traditional subtractive manufacturing. Selective laser melting (SLM) is a manufacturing technique gaining widespread popularity due to its unique benefits over conventional methods. This process involves the complete layer-by-layer melting of components under a protective atmosphere, resulting in precise and accurate production.

One of the most significant benefits is its low material consumption rate, resulting in considerable cost savings. Furthermore, SLM enables the creation of intricate and complex-shaped parts with minimal post-processing requirements [1,2]. This translates to a more efficient and streamlined manufacturing process, as less time and effort are spent on finishing the product. Overall, SLM is a highly effective and efficient manufacturing method that can significantly benefit businesses looking to optimise their production processes. SLM technology has gained wide popularity for manufacturing metal parts, such as steels [3], aluminium alloys [1,2,4,5], copper-based alloys [6], nickel-based alloys [7,8], titanium alloys [9], maraging steel [10,11], and super alloys [12].

Industries that require low-volume production for specific purposes, such as the medical, aeronautical, and mould sectors [13], are increasingly turning to AM technologies. However, the application of AM parts for structural purposes is presently limited because of uncertainties surrounding their mechanical properties. These mechanical properties are influenced by factors like porosity, internal defects, residual stresses, microstructural heterogeneities during the fabrication process [12,13], and the orientation of the construction, responsible for creating an anisotropic structure during AM [14].

Laser powder bed fusion (LPBF) is an AM technology that utilises a high-power laser beam to melt and consolidate metal powder layer by layer, creating intricate 3D objects. Due to the extreme thermal conditions, the printing process can give rise to transient phenomena and complex structural dynamics. The interplay between various factors often gives rise to structural anomalies, among which porosity is common. Keyhole porosity is a prevalent type due to the momentary collapse of the vapour depression zone [15,16,17,18].

Spattering is a phenomenon that occurs during laser powder bed fusion (LPBF) additive manufacturing. It is an intrinsic, unpreventable, and undesired event that can significantly impact the final product’s quality. Spattering can lead to metallurgical defects and the degradation of mechanical properties, particularly in the fabrication of large-sized parts during multi-laser LPBF. The formation of spatter can result in non-fusion defects, thereby negatively impacting the process stability, energy efficiency, and the quality of the manufactured objects. The mechanism behind spatter formation is attributed to hot spatter ejection, primarily driven by the Marangoni effect, and cold spatter ejection, mainly driven by the vapour-induced entrainment of the shielding gas. It is crucial to understand the spatter formation mechanism to mitigate the adverse effects of the spatter on the LPBF process [19].

In cases where there is an excess of laser energy input, specifically with a high power and slow scan velocity, metal vaporisation initiates a recoil pressure that exerts a downward force on the melt pool surface. This force generates a narrow, deep keyhole, within which several laser reflection and absorption events occur [20,21]. The non-uniform laser absorption in the surface creates a local hotspot and provokes an imbalance between recoil pressure, capillary force, and vapour dynamic pressure. In the context of unstable keyhole conditions, it has been observed that gas bubbles tend to pinch off at the keyhole tip. When these bubbles become trapped by the advancing solidification front, they may manifest as pore defects in the final product [17,18,22,23]. To minimise keyhole porosity in LPBF, the laser parameters are adjusted away from unstable zones. However, multiple variables in LPBF can induce local overheating and lead to keyhole porosity, even with optimised settings.

The rate at which heating and cooling occur has a significant effect on the residual stresses that often arise in SLM components. These stresses can lead to stress cracking and interlayer debonding. Typically, residual stresses result in high tensile stress at the top and bottom of the SLM sample and intermediate compression stress in the large intermediate zone [24].

The stress level in laser processing is mainly determined by the parameters used and can be managed by preheating the substrate before building to reduce the temperature gradient [25]. Furthermore, post-fabrication heat treatments (PFHTs) can eliminate or decrease residual stress levels.

Extensive research has been conducted on the fatigue resistance of specimens produced by 18Ni300 SLM [26,27], showing a poor fatigue performance of the as-built state of the parts [28,29,30]. This poor resistance arises due to metallurgical defects, inclusions, residual stress, and typical structures formed during SLM processes [31,32], which are caused by the construction parameters, surface roughness, and internal defects, the latter two being influenced by the former.

Inclusions and metallurgical defects can serve as initiators or triggers for cracks, decreasing fatigue resistance [33,34,35]. Based on current findings, fatigue cracks start from surface and internal defects. This leads to more instances of brittle fracture and reduced fatigue capabilities [28]. The introduction of inclusions containing titanium and aluminium oxides due to cross-contamination can significantly decrease the fatigue resistance of maraging steels produced through SLM [36].

Previous research [37,38] investigated the impact of the laser melting speed and porosity on the fatigue durability of sintered laser-melted steel components. The influence of post-manufacturing heat treatment on fatigue crack propagation has also been studied [30]. Additional investigation is required to ascertain how much the pore space generated by different building orientations influences the fatigue crack propagation.

Despite the widespread use of maraging steels in the mould industry and other applications, there is a lack of research on the relationship between AM printing strategies, porosity, and fatigue behaviour in 18Ni300 maraging steel. Additional research needs to be performed in order to increase the knowledge about the influence of porosity on the surrounding material and the pore space generated by different building orientations that influence the fatigue crack propagation.

## 2. Materials and Methods

The substrate material for SLM is a powder composed of 18Ni300 steel. The chemical composition provided by the manufacturer is presented in Table 1 for reference.

The powder was melted layer by layer using a high-power laser using the LPBF process to produce compact tension test (CT) specimens using Lasercusing^®^. The utilisation of Renishaw’s AM 400 equipment model in the LPBF process, coupled with the optimisation of the process parameters, resulted in the desired outcomes. A laser power of 400 W, a scan speed of 0.85 m/s, and a laser diameter of 0.04 mm were utilised. The deposited layer measured 30 µm in thickness, with a hatch spacing of 100 μm and a 25% overlap.

Tensile and yield strengths of 1147 MPa and 910 MPa, respectively, have been measured in the bulk material [39] for a 0° orientation. The density of the material is 7420 kg/m^3^, and the Vickers hardness is 352.7 HV. The Young’s modulus of the bulk material was determined through uniaxial tensile testing, and it yielded a value of 168 GPa. This value falls substantially below the typical benchmark of 210 GPa and is lower than the corresponding Young’s modulus for the wrought material [40]; Young’s modulus is influenced in AM by the post-heat treatment process [41]. Previously, there were noted differences between the wrought material and the additively manufactured material [27,42,43,44,45]. The degree of porosity in the samples, ascertained through building parameters, significantly impacts the observed discrepancy [39].

**Figure 1:**
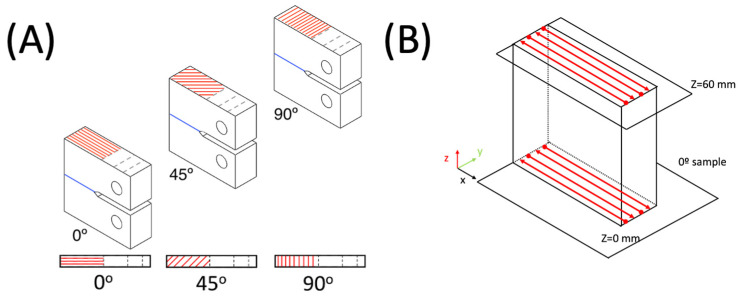
(**A**) SLM layer orientation pattern (red lines are weld bead orientation, blue lines are crack planes), and (**B**) SLM pattern of 0° sample starting on z = 0 mm and continuing layer by layer until z = 60 mm.

CT specimens were manufactured in SLM with different orientations, 0°, 45°, and 90°. The plane where these angles are printed is where, theoretically, the crack must propagate, and the angles are referred to as the direction of propagation of the crack, as shown in Figure 1A. The SLM followed the pattern shown in Figure 1B from z = 0 to z = 60 mm for each orientation, and the notch and the holes were machined.

Before commencing any testing procedures, the CT specimens were subjected to pre-cracking to ensure a more genuine crack pattern would occur during the testing process. Additionally, the surface was polished to augment the precision in determining the crack position. The surface roughness was measured and in terms of Ra was between 1.045 and 1.723 µm and in terms of Rq between 1.345 and 1.723 µm.

Five CT specimens were manufactured for each orientation using 18Ni300 maraging steel powder for casting. In order to conduct thorough testing, a single specimen was utilised for a full load test, while another was employed to calibrate the force necessary for fatigue cracking. The remaining three specimens were then dedicated to fatigue testing.

The experiments were conducted according to the ASTM E647 standard test method for determining the crack growth curve [46]. The Shimadzu AGS-X 50 kN in situ fatigue test machine was used to conduct fatigue crack initiation and propagation tests. A camera was employed as an additional data carrier to guarantee precise crack length measurements and accurate monitoring of its propagation.

Throughout the fatigue tests, a cyclic load spanning from 7 to 0.7 kN was consistently applied to each printed angle, with a rigorous fatigue stress rate of R = 0.1 being imposed. Load and displacement data were automatically collected every 0.01 s throughout each test. To ensure accurate determination of the crack length, precise measurements were taken using the MATLAB Image Viewer R2023b, with the distance between the crack tip and the back of the CT specimen being converted into pixel length.

Previously to the fatigue tests, the cross-section of each sample was examined in an optical microscope, Leica DM6 M. A specimen not used for fatigue testing was cut through two planes with constant Z (reference system Figure 1B) at a distance of approximately 10 mm in the central area of the specimen, i.e., at approximately Z = 35 mm and Z = 25 mm. These cuts were then polished to a mirror polish. In order to assess the porosity of various building orientations, visual imagery was acquired and subsequently subjected to surface enhancement techniques. This methodology incorporated a two-dimensional microscopic analysis to achieve accurate results, in accordance with other authors’ results [47]. 

Three main types of pores are thought to form during AM processes: keyhole pores, lack of fusion pores, and gas pores. The formation of keyhole pores is attributed to excessive energy input during the melting process. The penetration of metal powder is deepened by an excess beam power, creating a pore near the bottom of the melt pool after solidification. Typically, KH pores are characterised by a relatively large, circular shape in the horizontal plane and an elongated shape in the vertical plane. Lack of fusion pores occurs when there is a shortage of energy supplied to the powder bed during the melting process. Due to lower input energy, the metal powder fails to melt entirely, resulting in voids in the final structure. These pores are large, comparable to the melt pool, and irregular. Gas pores, the smallest and most spherical of all pores, are formed by trapped gas either already present in the metal powder or introduced during the melting process [47].

**Figure 2:**
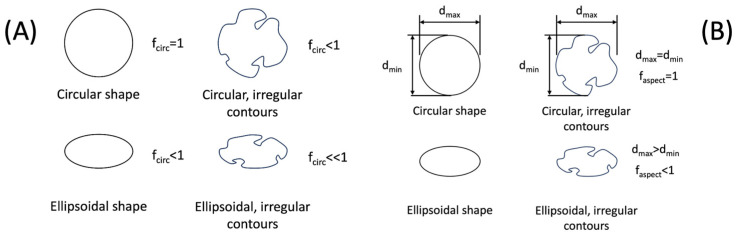
Example of how (**A**) circularity and (**B**) aspect ratio values are affected by different geometries and irregularities.

The size of the pores was determined using a pixel counting method using ImageJ 1.53t, a commercial software. Each pore was characterised by the circularity that represents the proximity to a circular cross-section:

(1) $$f_{circ.}=\frac{4\pi A}{p^{2}}$$

where *p* and *A* are the perimeter and the cross-sectional area of the pore, respectively. $f_{circ.}$ takes values in the interval ${0<f}_{circ.}\leq 1$, where 1 represents a perfectly circular cross-section (Figure 2A) [48].

The aspect ratio descriptor is given by

(2) $$f_{aspect}=\frac{d_{min}}{d_{max}}$$

$d_{max}\;and\;d_{min}$ are the maximum and minimum orthogonal diagonals of the pore. $f_{aspect}$ take values in the interval ${0<f}_{aspect}\leq 1,$ where a value away from unity refers to an elongation of the pore. The pore shape and its contour regularity level influence the shape factor, as shown in Figure 2B.

The present study encloses a comprehensive analysis of 120 individual pores concerning their circularity and aspect ratio in three distinct orientations. The ensuing data has been plotted against the pore diameter and area, with due consideration accorded to academic conventions and best practices. 

Microstructural analysis was conducted using scanning electron microscopy (SEM, CLARA-TESCAN, Brno, Czech Republic). Energy-dispersive X-ray spectroscopy (EDX) spot, line scan, and elemental mapping were employed to determine the chemical composition at cellular boundaries and between the welding beads. This technique enabled a semi-quantitative analysis, which allowed for an estimation of the chemical composition of non-metallic inclusions. Furthermore, the composition around porous areas was identified using this method.

In order to analyse the structure of the grain and grain boundaries in various orientations, the samples’ sections underwent a chemical treatment using Nital. This solution, which contained 4% nitric acid and ethyl alcohol, followed the ASTM E407 standard metallographic practice [49].

## 3. Results and Discussion

### 3.1. Metallography

**Figure 3:**
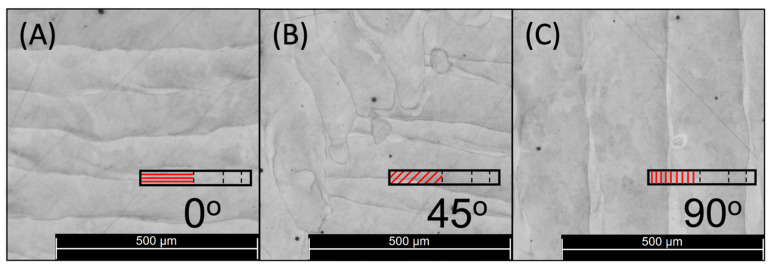
Optical micrograph of 18Ni300 maraging steel after being etched with Nital for 50 s at (**A**) 0°, (**B**) 45°, and (**C**) 90°.

The polished and etched specimens were examined through an optical micrograph to obtain the metallographic structure (Figure 3A–C). Figure 3A shows a 0° deposition track with some discontinuities typical of the melting process using a pulsating laser beam. The same is observed in Figure 3C, the 90° sample, where the track follows a straight line. Meanwhile, Figure 3B shows a 45° sample, formed as a combination of 0° and 90° welding beads, as shown in Figure 1A. Many melted pool boundaries (MPBs), which are inconsistent throughout the deposition process, are generated in this particular path. 

The impact of the pulsating laser beam on the melting process is well illustrated in Figure 3A–C. It is worth noting that the process exhibits a discontinuous nature across all the samples examined. The semi-elliptical shape is visible along the axis that is parallel to the building direction. Moreover, the overlapping of various scan tracks can be observed, which can be attributed to the imprecise nature of cutting machines [50]. The unique semi-elliptical shape exhibited by this object can be attributed to a thermal gradient that is particularly potent at the front edge of the laser beam. This gradient has a discernible impact on the growth of the scan track, ultimately leading to a cooler temperature at the front edge and resulting in a distinctive semi-elliptical shape. Across all samples, it has been observed that the width of the welding beads maintains a consistent measurement of 180 µm on average.

An uneven distribution of alloy elements in the specimens after they are built can be observed from the dark areas between the scan tracks. This phenomenon is caused by the high energy at the intersection points, which occurs when the laser beams overlap. The complex thermal processes involved in SLM create a cellular solidification structure that facilitates epitaxial growth across different track boundaries.

**Figure 4:**
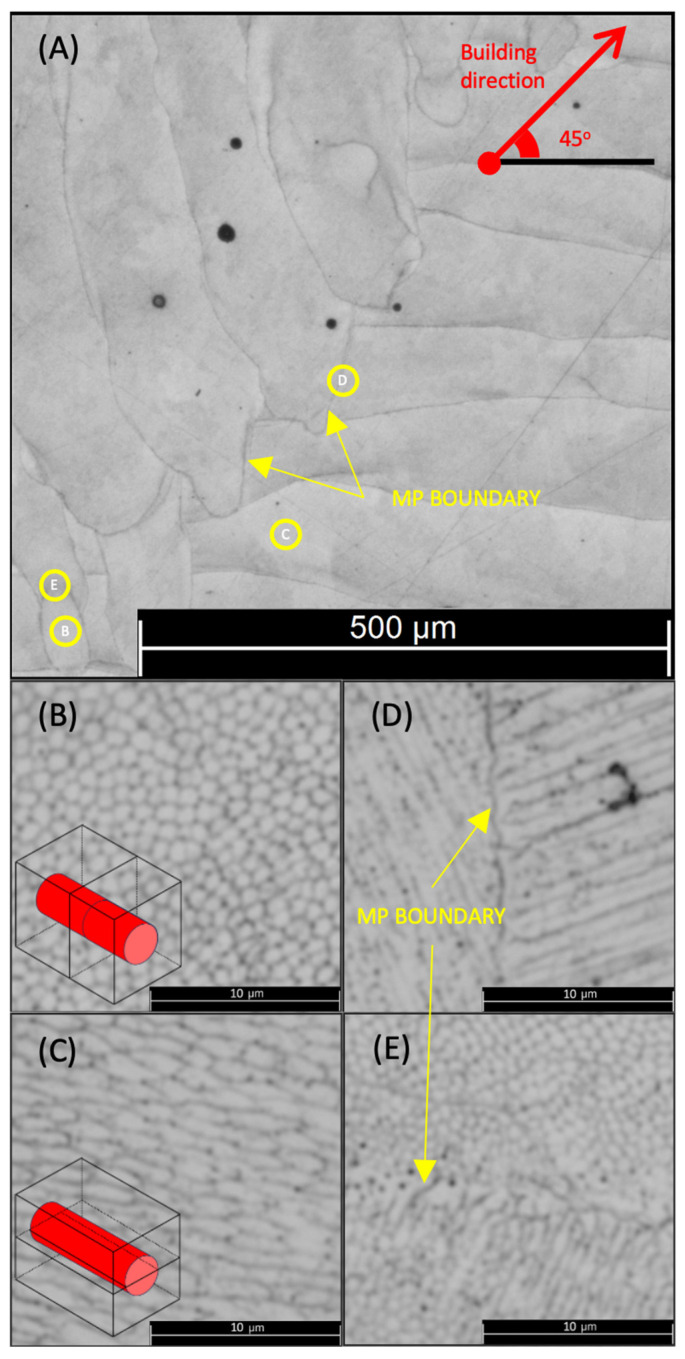
Optical micrographs of a 45° sample: (**A**) overview of the specimen; (**B**) vertical cross-section; (**C**) horizontal cross-section; (**D**) grain boundary between two horizontal cross-sections; and (**E**) grain boundary between horizontal and vertical cross-sections. Yellow arrows show the melted pool boundaries.

The cell distribution and size of the weld beads are shown in Figure 4B–E. Figure 4A shows an overview image of the weld beads of a 45° sample, showing the different structures in Figure 4B–E.

Upon a closer analysis of Figure 4B, it becomes evident that the constructed specimen possesses a vertical cross-section composed of a cellular microstructure that is submicron in size, with an intercellular spacing ranging from 1 to 1.5 μm. This distinct attribute is the fundamental reason behind the exceptional strength and hardness of the SLM maraging steel material.

Upon careful examination of the constructed sample, it was noted that the microstructure displayed small columnar and/or dendritic grains. These grains were a direct result of the rapid solidification of the melt pool, as indicated in Figure 4C. At high cooling rates, the formation of the α’ phase caused solidification and prevented the precipitation of intermetallic compounds. This resulted in specific components, such as Ni, Co, and Mo, remaining in a state of supersaturated solid solution [51].

Melted pool boundaries appeared between welding beads, showing a different microstructure on both welding beads. There are two primary morphologies on each side, small angle bunches consisting of strips and fine cellular structures. Figure 4D showcases a junction where two columnar structures with small angles intersect and share the same plane. Moreover, Figure 4E portrays the link between a cellular and columnar structure, providing further insight into the intricate details of the subject matter.

### 3.2. Defects in the Material

The defects in parts produced by LPBF are mainly caused by gases trapped in the melt and lack of fusion of the base material. However, imperfections due to lack of fusion were reduced due to the use of optimal manufacturing parameters. Although not null, they appeared much less frequently than pores caused by trapped gases, so only gas pores were considered.

**Figure 5:**
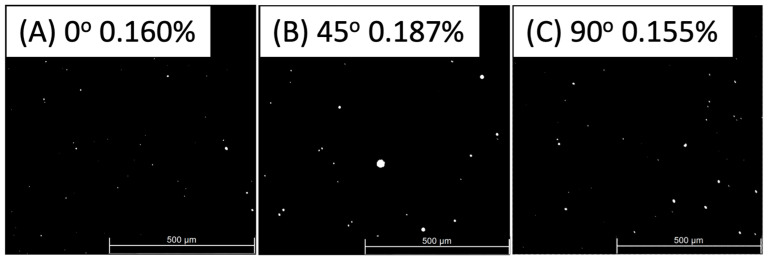
Porosity area surface in (**A**) 0°, (**B**) 45°, and (**C**) 90° samples obtained using Leica DM6 M, Wetzlar, Germany, and post-processed using ImageJ.

Upon thorough examination of the material, it was identified that the presence of pores is a crucial factor in the fatigue performance of the material. Our findings indicate that the extent of this defect is contingent upon the orientation of the samples being analysed. A visual representation is provided in Figure 5A–C to demonstrate this observation, which displays the percentage of porous area in each orientation. The data reveal that the 0° orientation exhibits a porous area of 0.160%, the 45° orientation presents 0.187%, and the 90° orientation shows 0.155%. Unsurprisingly, the 0° and 90° orientations exhibit a similar level of porosity, as their building strategy is similar (as shown in Figure 3A,C). However, the porosity increases when the 45° distribution is examined due to a greater number of grain boundaries, MPBs, which lead to the formation of pores.

**Figure 6:**
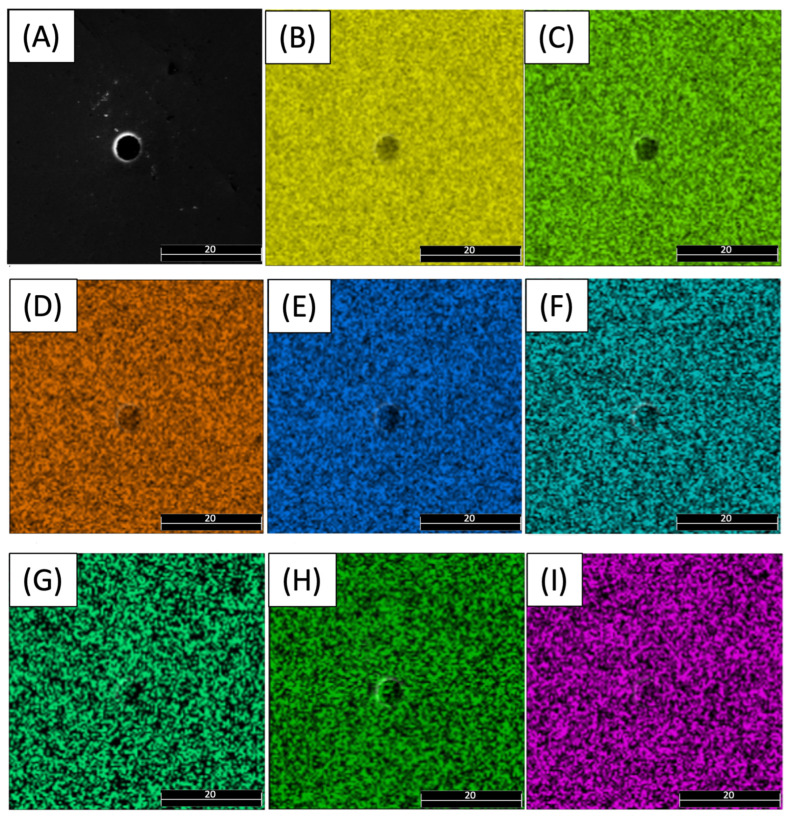
SEM and EDX images of a pore in 18Ni300 sample: (**A**) SEM image of a pore; element distribution of (**B**) Fe, (**C**) Ni, (**D**) Co, (**E**) Mo, (**F**) Ti, (**G**) Cu, (**H**) Al, and (**I**) Cr.

Figure 6 shows an SEM-EDX image that displays the various elements in the vicinity of a pore before any fatigue test. The elements identified in the image include Fe, Ni, Co, Mo, Ti, Cu, Al, and Cr, as denoted in Figure 6B–I. Upon close examination, it is apparent that these elements are evenly dispersed throughout the surrounding area of the pore. Thus, it can be inferred that the presence of the pore does not significantly influence the distribution of these elements.

**Figure 7:**
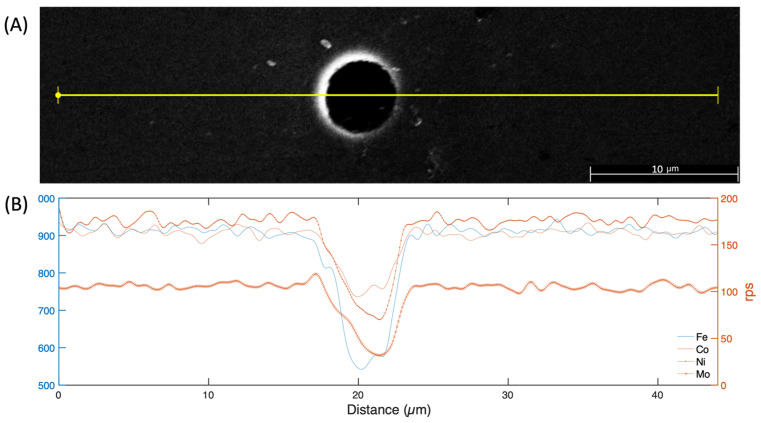
Linear EDX element analysis of the 18Ni300 sample through a pore: (**A**) SEM image and (**B**) element distribution.

In order to gain a more comprehensive insight into the chemical composition of a pore and detect any potential fluctuations in its environment, an EDX line scan was conducted. The findings obtained pertain to the same pore depicted in Figure 6 and are presented in Figure 7. During the analysis, an EDX line scan was performed on the pore’s interior and exterior sides. It is important to mention that the data collected inside the pore were not deemed significant and, therefore, were not considered for further analysis. Notably, only the matrix’s primary elements, Fe, Ni, Co, and Mo, were selected for thorough examination.

After disregarding the sensor’s full-scale perturbations and data acquisition time, the outcomes indicate a comparable concentration on either side of the pore, as shown in Figure 7B. The only variation is in the concentration of molybdenum, which is higher in the area where Fe, Ni, and Co are reduced, as seen in the left part of the pore that corresponds with the illuminated section of the SEM image in Figure 7A.

**Figure 8:**
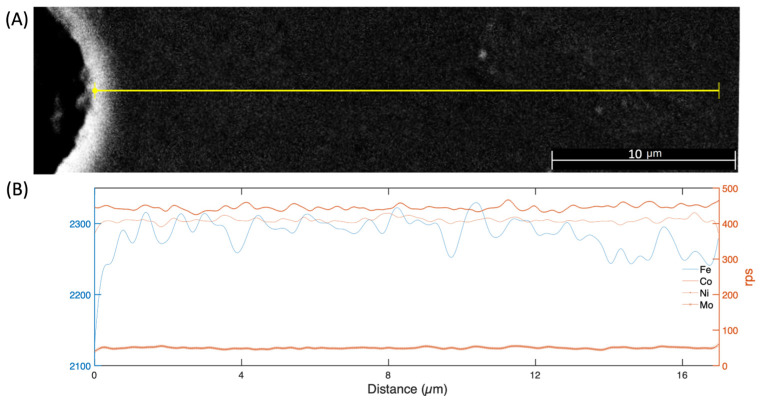
Linear EDX element analysis of the 18Ni300 sample from the boundary of a pore: (**A**) SEM image and (**B**) element distribution.

An examination was conducted using EDX to perform a line scan analysis on a single side of the pore (Figure 8). The findings revealed that all the essential elements displayed a uniform pattern without deviations. However, Fe presented a declining tendency proceeding away from the vicinity of the pore core, while Ni exhibited a minor rise. 

**Figure 9:**
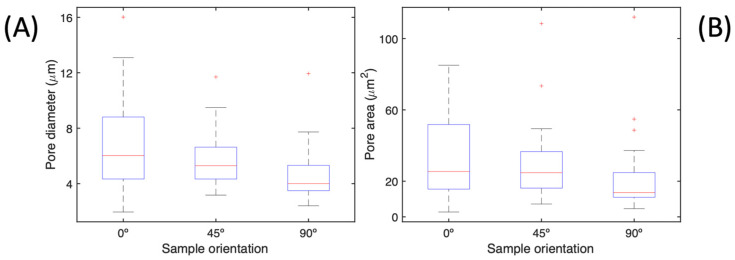
Porosity distribution in each orientation regarding the (**A**) pore diameter and (**B**) pore area.

A comprehensive overview of the pores analysed based on their orientation is presented in Figure 9, which includes the median and percentiles. Figure 9A highlights the distribution of pores based on their diameter, where a broader spread is observed at a 0° orientation. The data display comparable distributions at the 45° and 90° orientations. The median value for both orientations is within the 4–6 µm range. Figure 9B demonstrates a similar pattern, with the median pore area being approximately 20 µm^2^. As the angle increases from 0° to 90°, the distribution of pore area becomes less variable. The maximum pore area decreases from 80 µm^2^ at 0° to 35 µm^2^ at 90°, with a few outliers. The existence of any slight variations in pore diameter and area can be attributed to irregularities in the shape of the pore, such as circularity or aspect ratio other than 1.

It is important to mention that an equivalent diameter based on circular cross-sections has been used. Also, a minimum feature size threshold during image analysis has been set to guarantee that only pores above one pixel or 0.04 µm^2^ are counted. The purpose of this was to eliminate the impact of experimental noise at a low level, which cannot be avoided even with microscopic images. 

**Figure 10:**
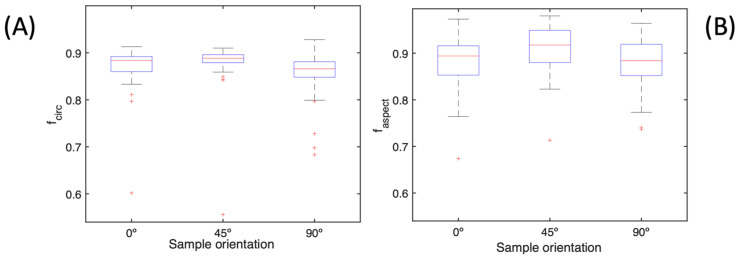
Arithmetic values of the (**A**) circularity factor and (**B**) aspect ratio.

Visual representations of the circularity and aspect factors for each orientation are displayed in Figure 10A,B. The circularity, shown in Figure 10A, has a median value of approximately 0.87 across all three orientations. Notably, at a 45° angle, there is a slight deviation from the median value, with most pores exhibiting circularity very similar to the median. When examining Figure 10B, it is evident that the aspect factor plays a significant role. Despite all orientations having a median of approximately 0.9, the variability is more important in this factor. The values are more dispersed, particularly for the 0° and 90° orientations. 

**Figure 11:**
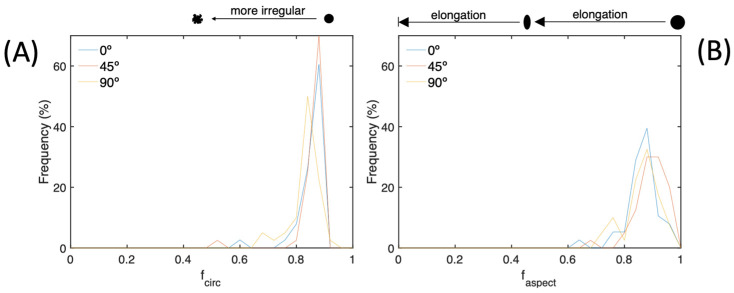
Frequencies in the 0°, 45°, and 90° specimens of the factor of (**A**) circularity and (**B**) aspect ratio of the pores.

Upon examining Figure 11A,B, it becomes evident that the circularity and aspect factors differ according to orientation. Specifically, our analysis reveals that the pores in question exhibit a high degree of circularity, with the majority falling within the range of 0.84–0.88. This range comprises a significant proportion (50–70%) of all the studied pores. It is consistent across all orientations, indicating that the pores are almost perfectly spherical and free from inclusions or irregularities. Observing Figure 11B closely, one can conclude that the pores are primarily clustered in the vicinity of the sphere without any inclusions. Nevertheless, they are not flawlessly shaped and appear to be slightly distorted. This is because most pores are positioned at levels surpassing 0.8 in every orientation. 

**Figure 12:**
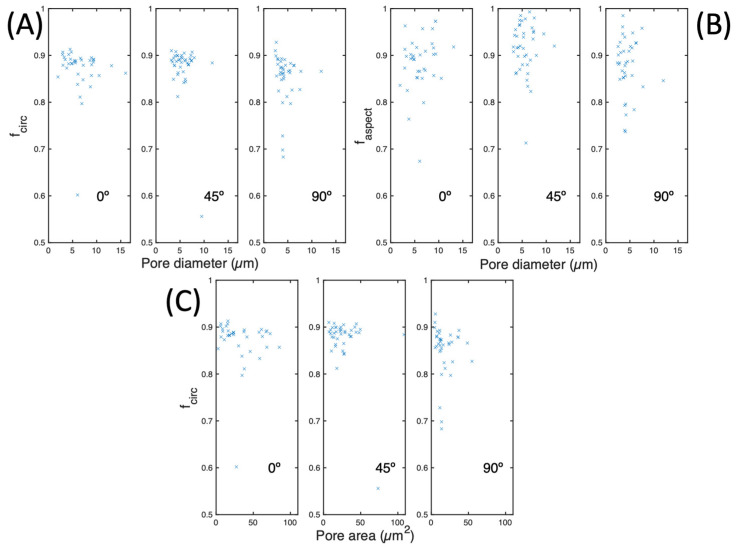
(**A**) Pore circularity, (**B**) pore aspect ratio as a function of pore diameter, and (**C**) pore circularity as a function of pore area in the 0°, 45°, and 90° samples.

The circularity ratio of the pores, as determined by their diameter and orientation, is depicted in Figure 12A. Each orientation exhibits a unique distribution of this factor, specifically, for 0° orientation, the distribution centres around 0.87 for pores of varying diameters. The circularity values for the 45° orientation are notably similar, with similar pore diameters. The 90° orientation, on the other hand, displays a broader distribution of factors, albeit with pore diameters below 10 µm. It is worth noting that hardly any pores in any orientation exceed a value of 0.9. Figure 12B displays the pore aspect ratio based on the pore diameter. The findings indicate that the 0° orientation has larger diameters than the 45° and 90° orientations, which are relatively evenly spread between 0.8 and 1. However, as validated in Figure 10B, the 45° orientation displays pore aspect ratios that are more uniform and closer to unity. The circularity ratio of the pores is presented in Figure 12C, plotted against their corresponding area. It can be observed that this graph exhibits a similar pattern to that of Figure 12A. 

### 3.3. Fatigue Test

**Figure 13:**
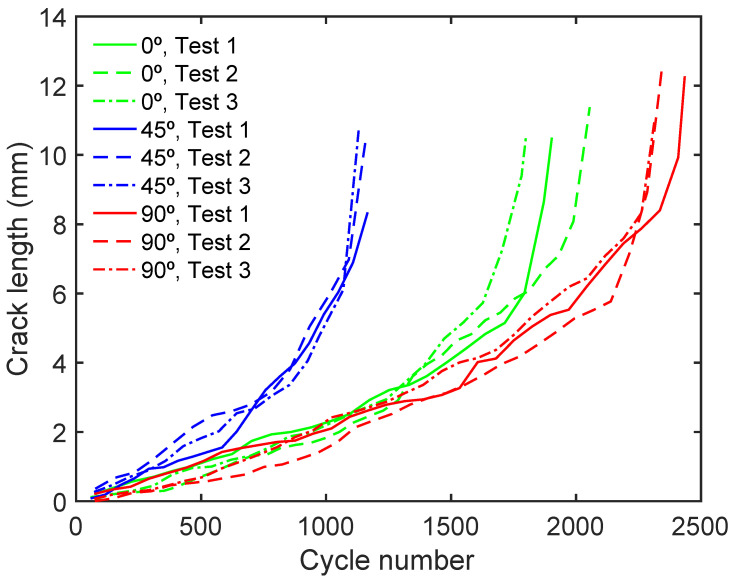
a/N curves for the as-built 18Ni300 SLM material: 0°, 45°, and 90° sample orientations.

To accurately calculate the fatigue life of a particular material, it is crucial to examine the crack growth plotted in the a vs. N graph, as shown in Figure 13, for samples taken at 0°, 45°, and 90° angles. Three samples were tested for each orientation, and interestingly, all orientations displayed consistent behaviour, with the three samples showing similar trends. According to the research findings, it is noteworthy that the 45° samples demonstrated a significantly higher crack growth rate for the same cycle number compared to the 0° and 90° orientations. Moreover, it was observed that the 45° samples had a life span that was half that of the 90° samples. Meanwhile, the 90° samples showed slower crack growth than the 0° samples.

Understanding the reasons behind the varying behaviours of samples fabricated under identical conditions is crucial. The microstructure differences are to blame, with the orientation of the welding beads resembling the melted pool boundaries where gas was trapped during fabrication, which led to pore formation in these areas, with differences between the 0°, 45°, and 90° samples.

While the composition of the pores may be alike, their existence can amplify the stress concentration factor and the likelihood of crack formation during a fatigue test. This can lead to elevated temperatures during the test and result in post-test segregation. Pores created by gaseous substances, which possess a high circularity and aspect ratio [47], may contribute to greater crack growth in specific orientations. Conversely, in this work, pores caused by insufficient fusion are infrequent and do not heavily impact this result, in contrast to previous research [52,53,54]. It is pertinent to note that the shape of a pore plays a decisive role in determining the fatigue behaviour of a material. Particularly, the aspect ratio of the pore, $f_{aspect}$, becomes increasingly deleterious at higher values, a trend evident from the data presented in Figure 13. Notably, samples featuring pores at a 45° angle exhibit a reduced lifespan owing to this factor and an increase in the porosity caused by the material deposition strategy in question.

## 4. Conclusions

In this research, a study of the porosity and fatigue behaviour of 18Ni300 SLM has been carried out with three main orientations (0°, 45°, and 90°) subjected to the same fabrication process. The following conclusions can be drawn:

1. An analysis of the element distribution around the pores has yielded a deeper understanding of the homogeneity of the material before subjecting it to fatigue testing. These findings indicate that the material exhibits uniform properties across all orientations that have been studied.
2. The sample manufacturing orientation directly influences its microstructure, as observed by a higher porosity with increased MPB. Notably, the 45° sample exhibited more MPB than the 0° and 90° samples, leading to a higher area and $f_{aspect}$. These findings underscore the importance of accounting for the manufacturing orientation when characterising the microstructure of samples (Figure 12B and Figure 13).
3. The fatigue behaviour of the specimens was evaluated by plotting the a/N curves of all samples. It was observed that the 45° sample tests exhibited a fairly inferior performance compared to the 90° and 0° tests. Specifically, the life span of the 45° specimens subjected to tests was found to be half that of the other two specimen orientations.

## Figures and Tables

**Table 1:** Chemical composition of 18Ni300 maraging steel.

wt/%	Fe	Ni	Co	Mo	Ti	Al	Cr	Cu	C
18Ni300	Balance	17–19	8.5–9.5	4.6–5.2	0.6–0.8	0.05–0.15	≤0.25	≤0.5	≤0.3

## Data Availability

Data are contained within the article.

## References

[1] Prashanth K.G., Scudino S., Klauss H.J., Surreddi K.B., Löber L., Wang Z., Eckert J. (2014). Microstructure and Mechanical Properties of Al–12Si Produced by Selective Laser Melting: Effect of Heat Treatment. Mater. Sci. Eng..

[2] Prashanth K.G., Shahabi H.S., Attar H., Srivastava V.C., Ellendt N., Uhlenwinkel V., Scudino S. (2015). Production of High Strength Al85Nd8Ni5Co2 Alloy by Selective Laser Melting. Addit. Manuf..

[3] Li R., Shi Y., Wang Z., Wang L., Liu J., Jiang W. (2010). Densification Behavior of Gas and Water Atomized 316L Stainless Steel Powder during Selective Laser Melting. Appl. Surf. Sci..

[4] Wang X.J., Zhang L.C., Fang M.H., Sercombe T.B. (2014). The Effect of Atmosphere on the Structure and Properties of a Selective Laser Melted Al–12Si Alloy. Mater. Sci. Eng..

[5] Louvis E., Fox P., Sutcliffe C.J. (2011). Selective Laser Melting of Aluminium Components. J. Mater. Process Technol..

[6] Gu D.D., Shen Y.F. (2006). Development and Characterisation of Direct Laser Sintering Multicomponent Cu Based Metal Powder. Powder Metall..

[7] Mumtaz K.A., Erasenthiran P., Hopkinson N. (2008). High Density Selective Laser Melting of Waspaloy^®^. J. Mater. Process Technol..

[8] Mumtaz K., Hopkinson N. (2009). Top Surface and Side Roughness of Inconel 625 Parts Processed Using Selective Laser Melting. Rapid Prototyp. J..

[9] Gu D., Hagedorn Y.C., Meiners W., Meng G., Batista R.J.S., Wissenbach K., Poprawe R. (2012). Densification Behavior, Microstructure Evolution, and Wear Performance of Selective Laser Melting Processed Commercially Pure Titanium. Acta Mater..

[10] Demir A.G., Previtali B. (2017). Investigation of Remelting and Preheating in SLM of 18Ni300 Maraging Steel as Corrective and Preventive Measures for Porosity Reduction. Int. J. Adv. Manuf. Technol..

[11] Rivalta F., Ceschini L., Jarfors A.E.W., Stolt R. (2021). Effect of Scanning Strategy in the L-PBF Process of 18Ni300 Maraging Steel. Metals.

[12] Amato K.N., Gaytan S.M., Murr L.E., Martinez E., Shindo P.W., Hernandez J., Medina F. (2012). Microstructures and Mechanical Behavior of Inconel 718 Fabricated by Selective Laser Melting. Acta Mater..

[13] Thompson S.M., Bian L., Shamsaei N., Yadollahi A. (2015). An Overview of Direct Laser Deposition for Additive Manufacturing; Part I: Transport Phenomena, Modeling and Diagnostics. Addit. Manuf..

[14] Shamsaei N., Yadollahi A., Bian L., Thompson S.M. (2015). An Overview of Direct Laser Deposition for Additive Manufacturing; Part II: Mechanical Behavior, Process Parameter Optimisation and Control. Addit. Manuf..

[15] Ren Z., Gao L., Clark S.J., Fezzaa K., Shevchenko P., Choi A., Everhart W., Rollett A.D., Chen L., Sun T. (2023). Machine Learning–Aided Real-Time Detection of Keyhole Pore Generation in Laser Powder Bed Fusion. Science.

[16] Martin A.A., Calta N.P., Khairallah S.A., Wang J., Depond P.J., Fong A.Y., Thampy V., Guss G.M., Kiss A.M., Stone K.H. (2019). Dynamics of Pore Formation during Laser Powder Bed Fusion Additive Manufacturing. Nat. Commun..

[17] Bayat M., Thanki A., Mohanty S., Witvrouw A., Yang S., Thorborg J., Tiedje N.S., Hattel J.H. (2019). Keyhole-Induced Porosities in Laser-Based Powder Bed Fusion (L-PBF) of Ti6Al4V: High-Fidelity Modelling and Experimental Validation. Addit. Manuf..

[18] Zhao C., Parab N.D., Li X., Fezzaa K., Tan W., Rollett A.D., Sun T. (2020). Critical Instability at Moving Keyhole Tip Generates Porosity in Laser Melting. Science.

[19] Li Z., Li H., Yin J., Li Y., Nie Z., Li X., You D., Guan K., Duan W., Cao L. (2022). A Review of Spatter in Laser Powder Bed Fusion Additive Manufacturing: In Situ Detection, Generation, Effects, and Countermeasures. Micromachines.

[20] Khairallah S.A., Martin A.A., Lee J.R.I., Guss G., Calta N.P., Hammons J.A., Nielsen M.H., Chaput K., Schwalbach E., Shah M.N. (2020). Controlling Interdependent Meso-Nanosecond Dynamics and Defect Generation in Metal 3D Printing. Science.

[21] Cunningham R., Zhao C., Parab N., Kantzos C., Pauza J., Fezzaa K., Sun T., Rollett A.D. (2019). Keyhole Threshold and Morphology in Laser Melting Revealed by Ultrahigh-Speed x-Ray Imaging. Science.

[22] Khairallah S.A., Anderson A.T., Rubenchik A., King W.E. (2016). Laser Powder-Bed Fusion Additive Manufacturing: Physics of Complex Melt Flow and Formation Mechanisms of Pores, Spatter, and Denudation Zones. Acta Mater..

[23] Wang Y., Guo W., Xie Y., Li H., Zeng C., Xu M., Zhang H. (2024). In-Situ Monitoring Plume, Spattering Behavior and Revealing Their Relationship with Melt Flow in Laser Powder Bed Fusion of Nickel-Based Superalloy. J. Mater. Sci. Technol..

[24] Guo L., Zhang L., Andersson J., Ojo O. (2022). Additive Manufacturing of 18% Nickel Maraging Steels: Defect, Structure and Mechanical Properties: A Review. J. Mater. Sci. Technol..

[25] Yves-Christian H., Jan W., Wilhelm M., Konrad W., Reinhart P. (2010). Net Shaped High Performance Oxide Ceramic Parts by Selective Laser Melting. Phys. Procedia.

[26] Damon J., Hanemann T., Dietrich S., Graf G., Lang K.H., Schulze V. (2019). Orientation Dependent Fatigue Performance and Mechanisms of Selective Laser Melted Maraging Steel X3NiCoMoTi18-9-5. Int. J. Fatigue.

[27] Croccolo D., De Agostinis M.A.S.S.I.M.I.L.I.A.N.O., Fini S., Olmi G., Vranic A., Ciric-Kostic S. (2016). Influence of the Build Orientation on the Fatigue Strength of EOS Maraging Steel Produced by Additive Metal Machine. Fatigue Fract. Eng. Mater. Struct..

[28] Branco R., Costa J.D.M., Berto F., Mohammad S., Razavi J., Ferreira J.A.M., Capela C., Santos L., Antunes F. (2018). Low-Cycle Fatigue Behaviour of AISI 18Ni300 Maraging Steel Produced by Selective Laser Melting. Metals.

[29] Hermann Becker T., Dimitrov D. (2016). The Achievable Mechanical Properties of SLM Produced Maraging Steel 300 Components. Rapid Prototyp. J..

[30] Santos L.M.S., Borrego L.P., Ferreira J.A.M., De Jesus J., Costa J.D., Capela C. (2019). Effect of Heat Treatment on the Fatigue Crack Growth Behaviour in Additive Manufactured AISI 18Ni300 Steel. Theor. Appl. Fract. Mech..

[31] Mercelis P., Kruth J.P. (2006). Residual Stresses in Selective Laser Sintering and Selective Laser Melting. Rapid Prototyp. J..

[32] Suryawanshi J., Prashanth K.G., Ramamurty U. (2017). Tensile, Fracture, and Fatigue Crack Growth Properties of a 3D Printed Maraging Steel through Selective Laser Melting. J. Alloys Compd..

[33] Zhu M.-L., Liu L.-L., Xuan F.-Z. (2015). Effect of Frequency on Very High Cycle Fatigue Behavior of a Low Strength Cr–Ni–Mo–V Steel Welded Joint. Int. J. Fatigue.

[34] Zhu M.-L., Xuan F.-Z., Du Y.-N., Tu S.-T. (2012). Very High Cycle Fatigue Behavior of a Low Strength Welded Joint at Moderate Temperature. Int. J. Fatigue.

[35] Zhu M.-L., Jin L., Xuan F.-Z. (2018). Fatigue Life and Mechanistic Modeling of Interior Micro-Defect Induced Cracking in High Cycle and Very High Cycle Regimes. Acta Mater..

[36] Gatto A., Bassoli E., Denti L. (2018). Repercussions of Powder Contamination on the Fatigue Life of Additive Manufactured Maraging Steel. Addit. Manuf..

[37] Santos L.M.S., Ferreira J.A.M., Jesus J.S., Costa J.M., Capela C. (2016). Fatigue Behaviour of Selective Laser Melting Steel Components. Theor. Appl. Fract. Mech..

[38] Santos L.M.S., Ferreira J.A.M., Costa J.D., Capela C. (2016). Fatigue Performance of Hybrid Steel Samples with Laser Sintered Implants. Procedia Eng..

[39] Cruces M.A.S., Crespo P.L., Branco R., Morales M.B.M., Borrego L.P. (2022). Propagación de Grietas de Fatiga Desde Concentrador En Acero Maraging Bajo Cargas de Tipo Biaxial. Rev. Española De. Mecánica De. La. Fract..

[40] ASM International (1991). ASM International Handbook Committee Properties and Selection: Irons Steels and High-Performance Alloys.

[41] Wu W., Wang X., Wang Q., Liu J., Zhang Y., Hua T., Jiang P. (2020). Microstructure and Mechanical Properties of Maraging 18Ni-300 Steel Obtained by Powder Bed Based Selective Laser Melting Process. Rapid Prototyp. J..

[42] Tan C., Zhou K., Tong X., Huang Y., Li J., Ma W., Kuang T. Microstructure and Mechanical Properties of 18Ni-300 Maraging Steel Fabricated by Selective Laser Melting. Proceedings of the 6th International Conference on Advanced Design and Manufacturing Engineering (ICADME 2016).

[43] Casalino G., Campanelli S.L., Contuzzi N., Ludovico A.D. (2015). Experimental Investigation and Statistical Optimisation of the Selective Laser Melting Process of a Maraging Steel. Opt. Laser Technol..

[44] Tariq F., Naz N., Baloch R.A. (2010). Effect of Cyclic Aging on Mechanical Properties and Microstructure of Maraging Steel 250. J. Mater. Eng. Perform..

[45] European Powder Metallurgy Association (2015). Introduction to Additive Manufacturing Technology, a Guide for Designers and Engineers.

[46] (2023). Standard Test Method for Measurement of Fatigue Crack Growth Rates.

[47] Snell R., Tammas-Williams S., Chechik L., Lyle A., Hernández-Nava E., Boig C., Panoutsos G., Todd I. (2020). Methods for Rapid Pore Classification in Metal Additive Manufacturing. JOM.

[48] Tammas-Williams S., Withers P.J., Todd I., Prangnell P.B. (2017). The Influence of Porosity on Fatigue Crack Initiation in Additively Manufactured Titanium Components. Sci. Rep..

[49] (2023). Standard Practice for Microetching Metals and Alloys.

[50] Mutua J., Nakata S., Onda T., Chen Z.C. (2018). Optimization of Selective Laser Melting Parameters and Influence of Post Heat Treatment on Microstructure and Mechanical Properties of Maraging Steel. Mater. Des..

[51] Davis J.R. (1998). Metals Handbook: Structure/Property Relationships in Irons and Steels.

[52] Walker K.F., Liu Q., Brandt M. (2017). Evaluation of Fatigue Crack Propagation Behaviour in Ti-6Al-4V Manufactured by Selective Laser Melting. Int. J. Fatigue.

[53] Chastand V., Quaegebeur P., Maia W., Charkaluk E. (2018). Comparative Study of Fatigue Properties of Ti-6Al-4V Specimens Built by Electron Beam Melting (EBM) and Selective Laser Melting (SLM). Mater. Charact..

[54] Majchrowicz K., Chmielewska A., Wysocki B., Przybysz-Gloc S., Kulczyk M., Garbacz H., Pakieła Z. (2023). The Effect of Microstructural Defects on High-Cycle Fatigue of Ti Grade 2 Manufactured by PBF-LB and Hydrostatic Extrusion. Crystals.

